# A quinazoline derivative suppresses B cell hyper-activation and ameliorates the severity of systemic lupus erythematosus in mice

**DOI:** 10.3389/fphar.2023.1159075

**Published:** 2023-05-15

**Authors:** Gan Zhang, Fan Yang, Juan Li, Shan Chen, Yuhang Kong, Chunfen Mo, Xiao Leng, Yang Liu, Ying Xu, Yantang Wang

**Affiliations:** ^1^ Clinical Laboratory, Clinical Medical College and the First Affiliated Hospital of Chengdu Medical College, Chengdu Medical College, Chengdu, China; ^2^ Department of Pharmacology, School of Pharmacy, Chengdu Medical College, Chengdu, China

**Keywords:** QNZ, B cell, systemic lupus erythematosus, activation, Kidney damage, quinazoline derivative

## Abstract

**Background:** Aberrant autoreactive B cell responses contribute to the pathogenesis of systemic lupus erythematosus (SLE). Currently, there is no safe and effective drug for intervention of SLE. Quinazoline derivative (N4-(4-phenoxyphenethyl)quinazoline-4,6-diamine, QNZ) is a NF-κB inhibitor and has potent anti-inflammatory activity. However, it is unclear whether QNZ treatment can modulate B cell activation and SLE severity.

**Methods:** Splenic CD19^+^ B cells were treated with QNZ (2, 10, or 50 nM) or paeoniflorin (200 μM, a positive control), and their activation and antigen presentation function-related molecule expression were examined by flow cytometry. MRL/lpr lupus-prone mice were randomized and treated intraperitoneally with vehicle alone, 0.2 mg/kg/d QNZ or 1 mg/kg/d FK-506 (tacrolimus, a positive control) for 8 weeks. Their body weights and clinical symptoms were measured and the frequency of different subsets of splenic and lymph node activated B cells were quantified by flow cytometry. The degrees of kidney inflammation and glycogen deposition were examined by hematoxylin and eosin (H&E) and PAS staining. The levels of serum autoantibodies and renal IgG, complement C3 deposition were examined by ELISA and immunofluorescence.

**Results:** QNZ treatment significantly inhibited the activation and antigen presentation-related molecule expression of B cells *in vitro*. Similarly, treatment with QNZ significantly mitigated the SLE activity by reducing the frequency of activated B cells and plasma cells in MRL/lpr mice.

**Conclusion:** QNZ treatment ameliorated the severity of SLE in MRL/lpr mice, which may be associated with inhibiting B cell activation, and plasma cell formation. QNZ may be an excellent candidate for the treatment of SLE and other autoimmune diseases.

## Introduction

Autoimmune diseases are caused by autoreactive B and T cells that attack body organs. Systemic lupus erythematosus (SLE) is one[ of the typical autoimmune diseases and often affects women in their child-bearing years. SLE usually has multiple B cell abnormalities, including dysregulation of B cell activation, an enlarged plasma cell population and elevated levels of high-affinity IgG and autoantibodies (Rupanagudi et al., 2015). The antigen/antibody immune complexes formed can deposit in the kidney and cause severe lupus nephritis (LN) (Wang et al., 2020). At present, the treatment of SLE remains complex, and there is the lack of safe, effective therapies for SLE. Therefore, the discovery of new therapeutic reagents will be of high significance in management of SLE patients.

Autoreactive B cell development and survival are regulated by both central and peripheral tolerance mechanisms (Karrar and Cunninghame Graham, 2018). Failure in autoreactive B cell tolerance contributes to the pathogenesis of SLE and LN (Yap and Chan, 2019). Functionally, B cells are critical for humoral and cellular immunity (Cai et al., 2019). A previous study has documented that autoreactive B cells participate in the pathogenesis of SLE by aberrant activation of naïve B cells, leading to the production of high levels of autoantibodies (Tomayko et al., 1950). Moreover, activated autoreactive B cells express high levels of MHC-II and costimulatory molecules to enhance their antigen presenting activity, contributing to the pathogenesis of SLE. Actually, novel therapeutic approaches to targeting the B cell repertoire include modulation of costimulatory signals in B-T cell interaction (Kambayashi et al., 1950; Perrigoue et al., 2009).

The nuclear factor kappa B (NF-κB) signaling is important for inflammation during the pathogenic process of SLE. Engagement of Toll-like receptor (TLR) or co-stimulator CD40 on B cells can activate the NF-κB signaling by promoting NF-κBp65 phosphorylation and IκBα degradation in naïve B cells and induce their proliferation (Yanagibashi et al., 2015; Robinson et al., 2019; Sun et al., 2020). Previous studies have shown that downregulation of MyD88 protein expression and NF-κB phosphorylation can ameliorate the severity of SLE (Liu et al., 2018; Wu et al., 2016; Zhan et al., 2021). QNZ, a chemical compound of N4-(4-phenoxyphenethyl)quinazoline-4,6-diamine, is a potent NF-κB inhibitor (Zhu et al., 2020), and has strong anti-aging, anti-inflammatory and antitumoral activity (Armstrong et al., 2008; Zhu et al., 2020). Treatment with QNZ can delay aging process by reducing chondrocyte degeneration (Zhu et al., 2020), relieve carrageenan-induced foot swelling and inhibit the growth of tumors in rodents (Marciano et al., 2019). However, little is known about whether treatment with QNZ can modulate B cell activation and survival as well as the pathogenic process of SLE.

In this study, we studied the potential effects of QNZ on B cell function and SLE in mice. Our data indicate that QNZ inhibits B cell activation and antigen presentation activity *in vitro*. Furthermore, treatment with QNZ alleviated the severity of SLE in MRL/lpr mice by inhibiting B cell activation, reducing serum IgG/autoantibody levels and mitigating kidney damage.

## Materials and methods

### Reagents and antibodies

**FIGURE 1:**
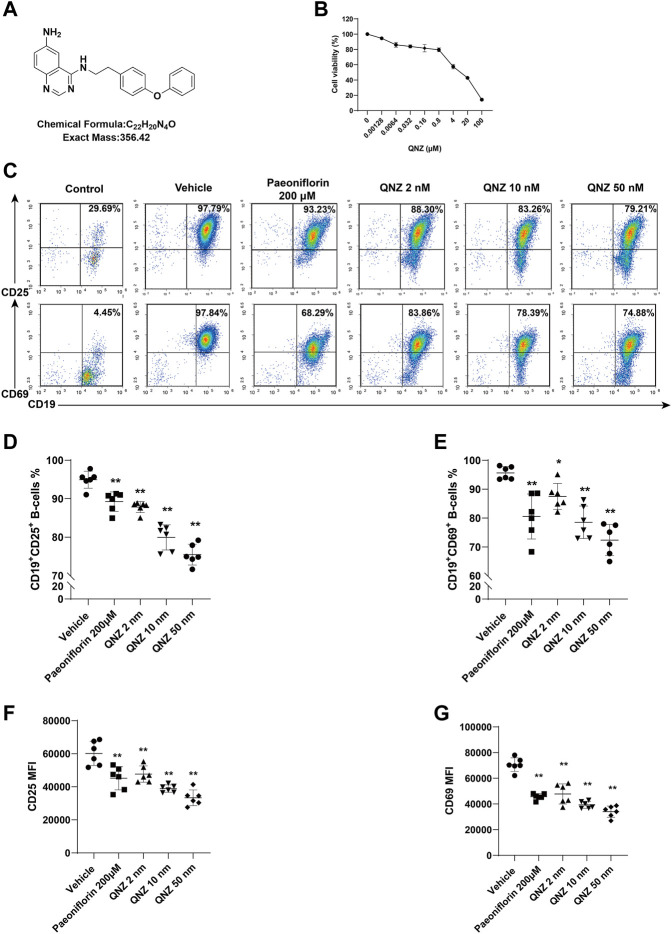
QNZ inhibits B cell activation *in vitro*. Splenic B cells were purified from 6–8 weeks of age mice and treated with vehicle or the indicated concentrations of QNZ to measure the potential cytotoxicity of QNZ by CCK-8 assays. Furthermore, the purified B cells were pretreated with vehicle control (0.1% DMSO) or the indicated doses of QNZ or paeoniflorin, followed by measuring the frequency of activated B cells by flow cytometry. **(A)** The structure of QNZ. **(B)** The viability of B cells following treatment with the indicated drugs for 18 h **(C–E)** Flow cytometry analysis of activated B cells. “Control” group: unstimulated B cells. **(F, G)** The levels of CD25 and CD69 expression on B cells. The data are expressed as the mean ± SD from five to six independent experiments and were analyzed by one-way ANOVA. *p < 0.05, **p < 0.01.

Special antibodies included FITC-conjugated anti-CD83 (Michel-19 clone, 121506), Alexa Fluor 647-conjugated anti-CD80 (16-10A1 clone, 104718), BV650-conjugated anti-CD86 (GL-1 clone, 105035), BV421-conjugated anti-I-A/I-E (MHC-II) (M5/114.15.2 clone, 107631), PE-conjugated anti-CD40 (3/23 clone, 124610), PE-cy7-conjugated anti-CD19 (6D5 clone, 115520), PE-conjugated anti-CD19 (6D5 clone, 115508), PE-conjugated anti-CD69 (H1.2F3 clone, 104508), BV421-conjugated anti-CD25 (PC61 clone, 102033), APC-conjugated anti-CD138 (281-2 clone, 142506), APC-conjugated anti-CD1d (1B1 clone, 123522), PE-cy7-conjugated anti-CD5 (53-7.3 clone, 100622), FITC-conjugated anti-CD21 (7E9 clone, 123408), Alexa Fluor 647-conjugated anti-CD23 (B3B4 clone, 101612) and purified anti-mouse IgG1 (RMG1-1 clone, 406601) (Biolegend, San Diego, United States). The FITC Annexin V Apoptosis Detection Kit with 7-AAD was purchased from BD PharMingen (San Diego, United States). The rat mAb against C3 was purchased from Abcam (Cambridge, United States). Other special reagents included 5 (6)-carboxyfluorescein diacetate succinimidyl ester (CFSE), Alexa Fluor 594 goat anti-mouse IgG (H + L) and Alexa Fluor 594 goat anti-rat IgG (H + L) (Invitrogen, Waltham, United States), mouse IgG ELISA kit, mouse ANA ELISA kit and mouse dsDNA ELISA kit (Mlbio, Shanghai, China), QNZ (Selleck, Houston, United States), paeoniflorin (PF) and FK-506 (tacrolimus) (CNS Pharm, Houston, United States). The molecular structure of QNZ is shown in Figure 1A. The PF was dissolved in PBS and other chemical compounds were dissolved in dimethyl sulfoxide (DMSO).

### B cell purification and viability

Female C57BL/6 mice at 6–8 weeks of age were euthanized and their splenic CD19^+^ B cells were purified by negative selection using a B cell isolation kit (Miltenyi Biotec, Germany, 130-090–862). The purity of the CD19^+^ B cells was above 95%. The purified CD19^+^ B cells (2 × 10^5^ cells/well) were treated in triplicate with different concentrations of QNZ in 96-well plates in RPMI 1640 supplemented with 10% fetal bowel serum (FBS, Gibco, United States), 1% of penicillin and streptomycin (Hyclone, United States) at 37°C in 5% CO2 incubator for 18 h. During the last 6-h culture, each well of the cells was added with 10 µL CCK-8 solution. The cell viability in each well was measured for absorbance at 450 nm.

### B cell activation and antigen presentation function *in vitro*

The purified CD19^+^ B cells were pre-treated with 0.1% DMSO (vehicle), QNZ (2 nM, 10 nM, or 50 nM) or PF (200 μM, a positive control) for 2 h. The cells were stimulated with 10 μg/mL of lipopolysaccharides (LPS, Sigma-Aldrich, St Louis, United States), 1 μg/mL of CD40L and 10 ng/mL of IL-4 (PeproTech, Cranbury, United States) for 18 h. The cells were stained with PE-cy7-anti-CD19, BV421-anti-CD25, PE-anti-CD69 and PE-cy7-anti-CD19, FITC-conjugated anti-CD83, Alexa 647-conjugated anti-CD80, BV650-conjugated anti-CD86, BV421-conjugated anti-I-A/I-E (MHC-II), PE-conjugated anti-CD40. The activation and antigen presentation function of B cells were analyzed by flow cytometry in a Novocyte flow cytometer (ACEA Bioscience, China). Annexin V staining was used to select viable cells for flow cytometry.

### Mice and drug treatment

Female C57BL/6 mice at 6–8 weeks of age for the experiment *in vitro* and female BALB/c mice at 6–8 weeks of age for the experiment *in vivo* were obtained from the Shanghai Model Organisms (China). Female MRL/lpr at 7–8 weeks of age for the experiment *in vivo* were purchased from Covance (Shanghai, China). All mice were bred and housed under a specific pathogen-free (SPF) condition in the experimental animal center of Chengdu Medical College at consistent room temperatures (20°C–26°C) and humidity (40%–70%). All mice were provided with normal food and water. All animal protocols were approved by the Animal Care and Use Committee of Chengdu Medical College (Approval NO. CMC-IACUC-2021021).

The mice were randomized and injected intraperitoneally with 0.2 mg/kg/d QNZ in the solvent of 1% DMSO, 8% PEG300, 1% Tween80% and 90% saline, 1 mg/kg/d FK-506 (a positive control, tacrolimus) in the solvent of 5% DMSO, 2% Tween80% and 93% saline or the solvent alone (1% DMSO, 8% PEG300, 1% Tween80% and 90% saline) daily for 8 weeks. Their body weights were measured weekly. The mice were euthanized at 17 weeks of age and their spleen and kidney tissues were dissected and measured. The spleen index (spleen weight/body weight × 100%) and kidney index (kidney weight/body weight × 100%) were calculated. The sizes of spleens and lymph nodes in each group of mice were compared.

### Flow cytometric analysis

Splenic and lymph node mononuclear cells were stained with a combination of multiple mAbs against surface markers in the dark for 30 min on ice. The fluorescent mAbs for B cell activation were PE-cy7-anti-CD19, BV421-anti-CD25 and PE-anti-CD69. The fluorescent mAbs for plasma cells were PE-cy7-anti-CD19 and APC-CD138. The fluorescent mAbs for marginal zone B (MZ-B)/follicular B (FO-B) and B1 cells included PE-cy7-anti-CD19, FITC-anti-CD21, APC-anti-CD23 and PE-anti-CD19, PE-cy7-anti-CD5, APC-anti-CD1d, respectively. FMO controls were used for activation marker assessment.

### ELISA and anti-nuclear Ab assays

The levels of serum IgG, ANA and anti-dsDNA in individual MRL/lpr were quantified using mouse IgG ELISA kit, ANA ELISA kit and dsDNA ELISA kit. The serum samples were diluted at 1:2 and the control and experimental serum samples were tested in triplicate simultaneously. The absorbance was measured at 450 nm using a microplate reader (Bio Tek, United States).

### Histopathology and immunofluorescence analysis of MRL/lpr mice

The mice were anesthetized with 2% pentobarbital sodium (50 mg/kg) and perfused with saline and then with 4% (w/v) paraformaldehyde. Their kidneys were dissected and embedded in paraffin. The kidney tissue sections (5 µm) were stained with H&E and PAS (Beyotime, China), respectively. Images were captured using an OLYMPUS BX63 (Japan).

The mice were anesthetized and perfused with saline and then with 4% (w/v) paraformaldehyde. Their kidney tissues were embedded in optimum cutting temperature (OCT) (SAKURA, America) and frozen in dry ice. The crystal kidney tissue sections (20 µm) were stained with anti-mouse IgG1 (1: 50) or rat mAb against mouse complement 3 (C3, 1: 50), followed by staining with Alexa Fluor 594 goat anti-mouse IgG (H + L) (1: 200) or Alexa Fluor 594 goat anti-rat IgG (H + L) (1: 200), respectively. After being washed, the red fluorescent signals were captured using an OLYMPUS BX63.

Blinded scoring of kidney severity on a scale from 0 (normal) to 4 (end stage) was based on the presence and severity of glomerular basement membrane, sacculus adhesion, interstitial fibrosis and glycogen deposition. Image-Pro Plus software was used to analyze the kidney interstitial inflammatory infiltration.

### Data and statistical analysis

Data are expressed as mean ± SD. The difference among groups was analyzed by repeated analysis of variance (ANOVA) and *post hoc* Bonferroni correction or nonparametric Kruskal-Wallis tests (no Gaussian distribution) using PRISM software (version 8.0; GraphPad Software). Statistical significance was set at *p < 0.05, **p < 0.01.

## Results

### QNZ inhibits mouse B cell activation *in vitro*

Naïve B cells can be activated and the activated B cells can differentiate into antibody-producing plasma cells. The aberrant B cell activation can contribute to the development of SLE. First, we tested the impact of different concentrations of QNZ on the viability of B cells *in vitro*. Splenic B cells were purified from healthy C57BL/6 mice and treated with the indicated concentrations of QNZ for 18 h and their viability was determined by CCK-8 assays. As shown in Figure 1B, treatment with QNZ at a range of low doses did not significantly affect the viability of B cells, but treatment with QNZ at a dose of >4 µM significantly decreased the viability of B cells *in vitro* (Figure 1B). Accordingly, we chose much lower doses of QNZ for our subsequent experiments. Next, we tested whether treatment with QNZ could modulate B cell activation *in vitro*. The purified B cells were pretreated with QNZ (2 nM, 10 nM or 50 nM), PF (200 μM) (Zhang et al., 2021; Zhang and Wei, 2020) or vehicle control (0.1% DMSO) for 2 h. The cells were stimulated with 10 μg/mL of LPS, 1 μg/mL of CD40L and 10 ng/mL of IL-4 for 18 h to activate B cells. Following being stained with fluorescent antibodies against CD25 or CD69, the percentages of CD19^+^CD25^+^ or CD19^+^CD69^+^ activated B cells were quantified by flow cytometry (Figure 1C). Compared to the inactive group (Control), the B cells in the vehicle control group (Vehicle) were significantly activated. Treatment with PF significantly decreased the frequency of activated B cells, and treatment with QNZ also significantly reduced the percentages of activated B cells in a dose-dependent manner (Figures 1C–E). A similar pattern of CD25 and CD69 expression was detected in different groups of B cells (Figures 1F, G). Hence, treatment with QNZ significantly inhibited B cell activation in our experimental conditions.

### QNZ inhibits the expression of antigen presenting-related molecules of activated B cells *in vitro*

**FIGURE 2:**
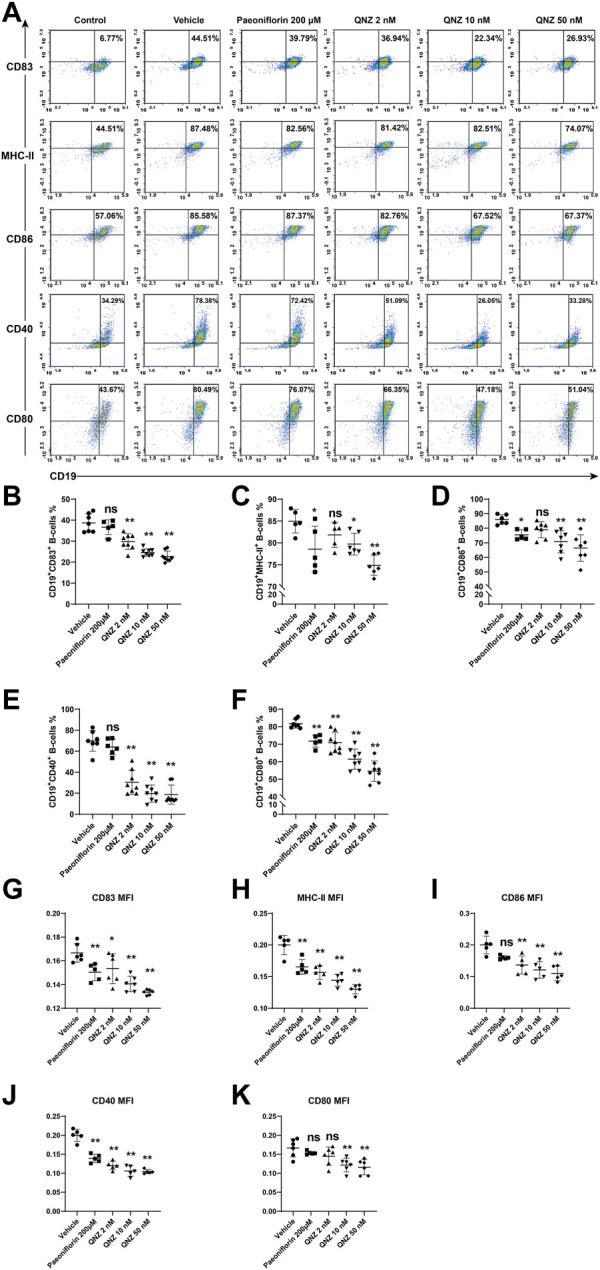
Flow cytometry analysis of the expression of molecule-related to antigen presenting activity in activated B cells. Following stimulation of B cells with 10 μg/mL of LPS, 1 μg/mL of CD40L and 10 ng/mL of IL-4 for 18 h in the presence or absence of the indicated compound, the expression of CD83, MHC-Ⅱ, CD86, CD40, and CD80 in B cells was analyzed by flow cytometry after staining with fluorescent antibodies. **(A)** Representative flow cytometry charts. “Control” group: unstimulated B cells. **(B–F)** Quantitative analysis of the frequency of each subset of B cells. **(G–K)** The levels of each co-stimulator tested. Using the normalization method to process group “Vehicle”. The data are representative flow cytometry dot plots or expressed as the mean ± SD from five to six independent experiments and were analyzed by one-way ANOVA. Normalized the MFI of the “Vehicle” group. *p < 0.05, **p < 0.01.

Activated B cells act as one type of the professional antigen presenting cells and can induce T cell activation by expressing high levels of MHC-II and co-stimulating molecules, such as CD80, CD86, CD40, and CD83 (Kambayashi et al., 1950; Perrigoue et al., 2009). Accordingly, we examined the effect of QNZ on the expression of these molecules on B cells following activation. Treatment with PF significantly decreased the percentages of CD19^+^CD80^+^, CD19^+^MHC-II^+^, CD19^+^CD86^+^ B cells, but not CD19^+^CD83^+^ and CD19^+^CD40^+^ B cells (Figures 2A–F). Treatment with QNZ also significantly reduced the frequency of these subsets of B cells, particularly for a higher dose of QNZ. Furthermore, treatment with QNZ significantly decreased the levels of CD83, CD80, CD86, CD40, and MHC-II expression on B cells and its inhibitory effects were greater than that of PF (Figures 2G–K). The significantly decreased expression of antigen presenting-related molecules indicated that QNZ treatment inhibited the antigen presenting activity of activated B cells.

### Treatment with QNZ attenuates SLE severity in MRL/lpr mice

**FIGURE 3:**
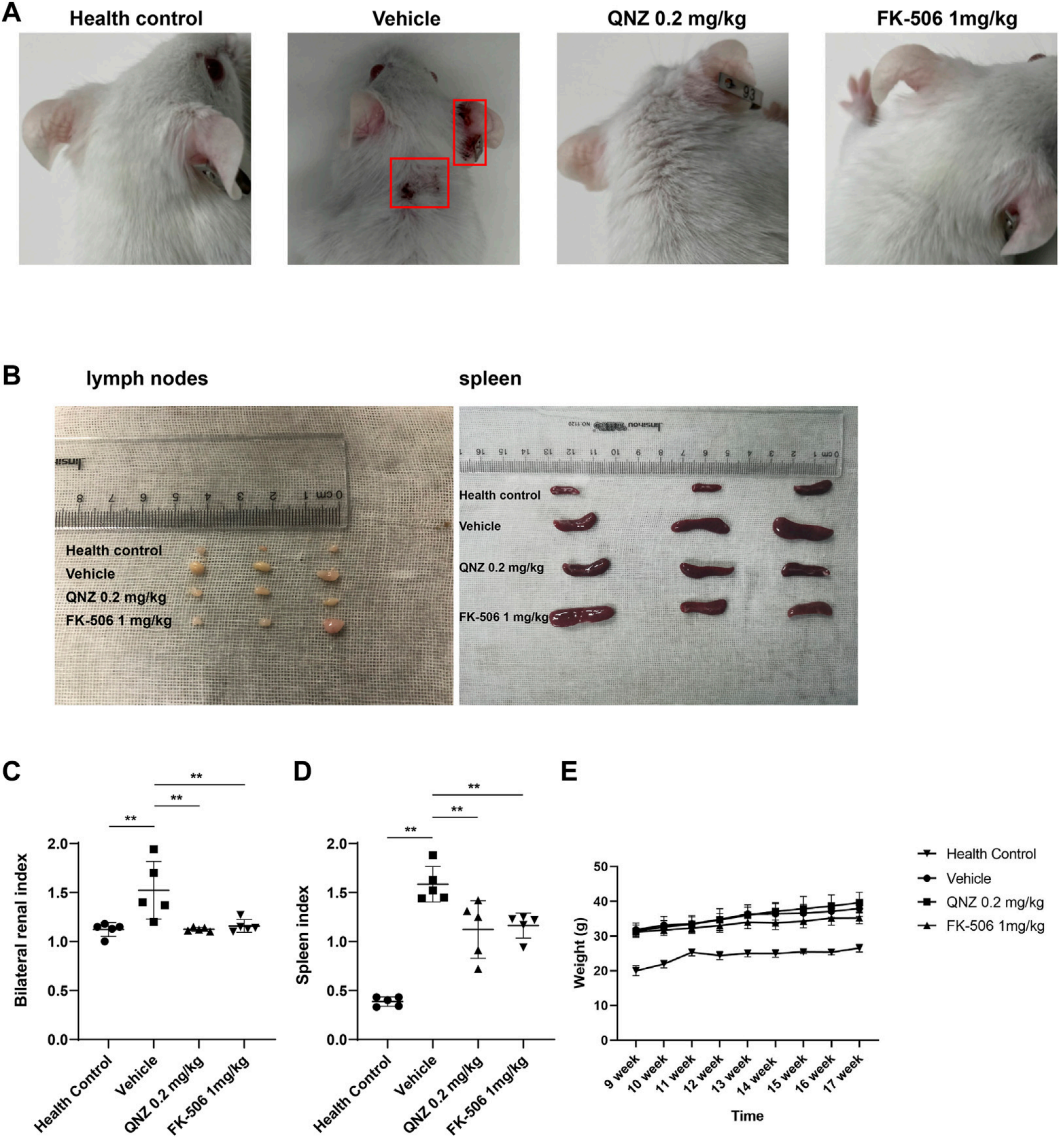
Treatment with QNZ ameliorates clinical symptoms in MRL/lpr mice. Female MRL/lpr mice were randomized and injected intraperitoneally with vehicle control, the indicated dose of QNZ or FK-506 daily for 8 weeks. Their body weights were measured longitudinally. At the end of the experiment, their skin lesions were evaluated and their lymph nodes and spleens were dissected and photoimaged, Furthermore, their kidneys and spleens were weighed and their indexes were calculated. **(A)** Representative images of skin ulcers in mice. **(B)** The spleen and lymph node sizes in mice. **(C, D)** The values of spleen index and kidney index. **(E)** The body weights of each group of mice. Female BALB/c mice of the same age as health control. The data are representative images or expressed as the mean ± SD from each group (*n* = 5, 6) of mice and were analyzed by one-way ANOVA. *p < 0.05, **p < 0.01.

To explore the therapeutic effect of QNZ on the severity of SLE, female MRL/lpr mice were randomized and treated with vehicle control, QNZ (0.2 mg/kg/d) or FK-506 (1 mg/kg/d) for 8 weeks. Compared with the vehicle-treated mice, treatment with QNZ or FK-506 alleviated skin ulcers (Figure 3A) and reduced the hypertrophy of spleens and lymph nodes in mice (Figure 3B). Measurement of spleen and kidney weights indicated that treatment with QNZ, like FK-506, significantly mitigated the gains of kidney index and spleen index in mice (Figures 3C, D). However, there was no significant change in the body weights of different groups of SLE mice (Figure 3E) and there was no dead animal throughout the observation period. Collectively, treatment with QNZ, like FK-506, significantly mitigated the SLE-related clinical symptoms in female MRL/lpr mice.

### Treatment with QNZ attenuates kidney damages in MRL/lpr mice

**FIGURE 4:**
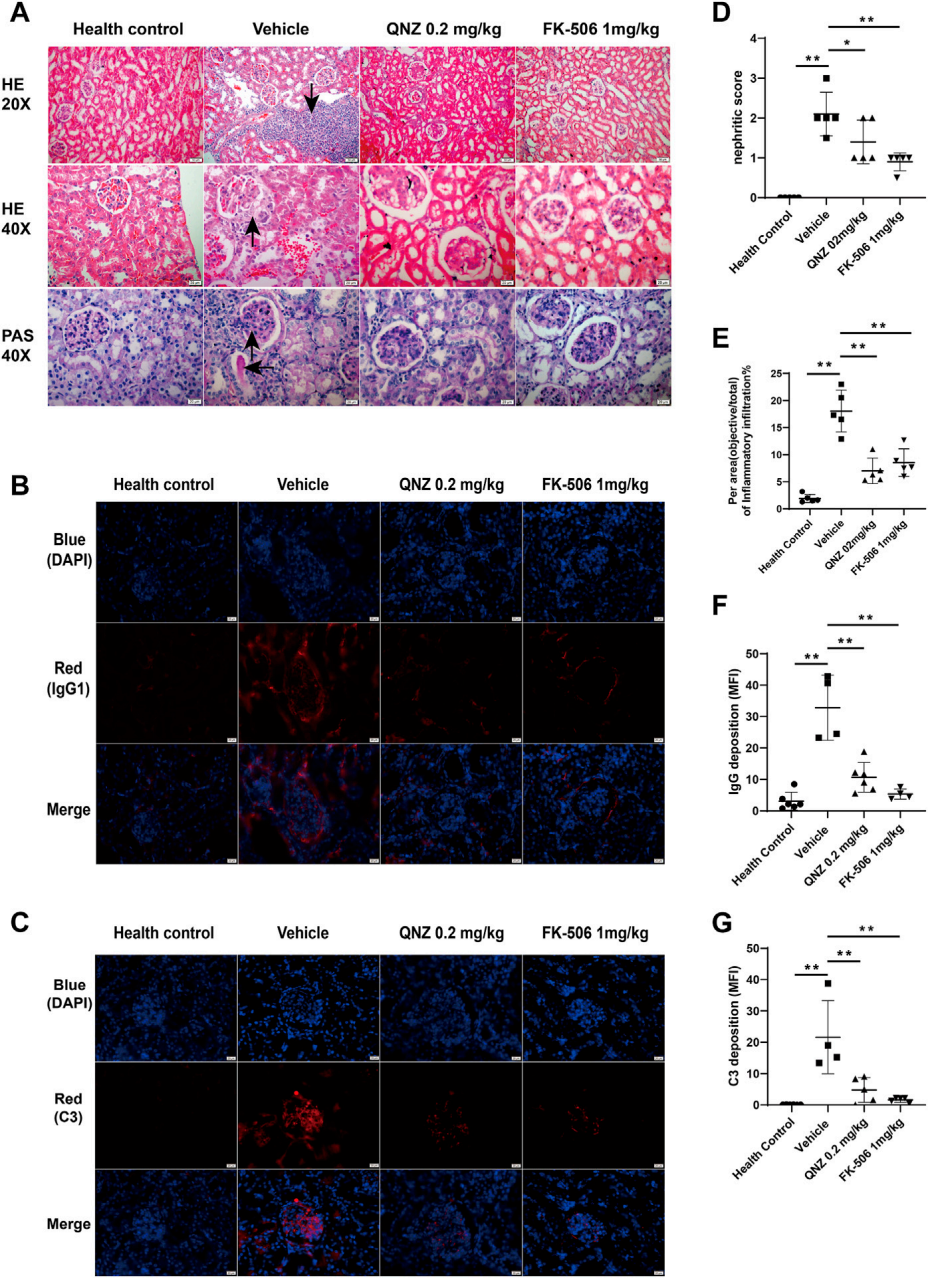
Treatment with QNZ reduces renal tissue damages and IgG and complement 3 depositions in the kidney tissues of mice. Following treatment with QNZ or FK-506 for 8 weeks, the mice were perfused and euthanized. Their kidney tissues were dissected and the paraffin-embedded kidney tissue sections were stained with H&E and PAS. In addition, the kidney tissues were embedded in OCT and frozen, and their crystal sections were stained with fluorescent antibodies against mouse IgG or C3 (red color), followed by nuclearly staining with DAPI. **(A)** H&E and PAS staining of kidney tissue sections. Scale bar: 20 μm. **(B, C)** Immunofluorescent analysis of IgG1 and C3 deposition in the kidney tissues. Scale bar: 20 μm. **(D)** Different groups of mice were scored for glomerular abnormalities on a scale from 0 to 4. **(E)** The per area (objective/total×100%) of inflammatory infiltration in H&E staining of kidney section. **(F, G)** Mean fluorescence intensity (MFI) of IgG and C3 staining. The data are representative images from each group of mice. The data are expressed as the mean ± SD. (*n* = 4–6 independent experiments/group) *p < 0.05, **p < 0.01.

Kidney damage is one of the serious symptoms in SLE. The presence of LN significantly increases the risk of renal failure and patient mortality. The LN is characterized by the deposition of immune complex in the basement membrane of the glomerular microvessels, resulting in activation of the alternative complement pathway (Gottschalk et al., 2015). We further analyzed kidney tissues after H&E and PAS staining and found that there were many inflammatory infiltrates and great glycogen deposition in the kidney (Figure 4A). While the vehicle-treated MRL/lpr mice displayed severe glomerular injury, interstitial inflammation, glycogen deposition and extensive intraglomerular IgG1 and C3 deposition in kidney tissues, the QNZ-treated mice exhibited the reduced degrees of glomerular basement membrane thickening and sacculus adhesion, interstitial inflammatory infiltration, interstitial fibrosis and glycogen deposition (Figures 4A, D,E). Immunofluorescence of kidney sections revealed lower IgG1 and C3 deposition in the glomeruli of the QNZ-treated mice, related to that the vehicle-treated MRL/lpr mice (Figures 4B,C, F,G). Thus, treatment with QNZ, like FK-506, dramatically mitigated the severity of LN in MRL/lpr mice.

### Treatment with QNZ decreases the frequency of activated B cells in MRL/lpr mice

**FIGURE 5:**
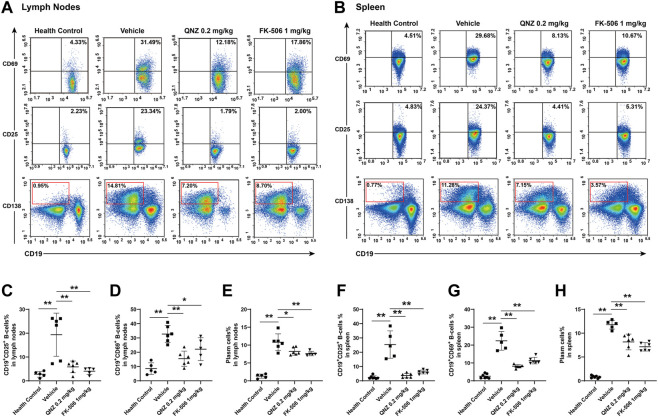
Flow cytometry analysis of the frequency of activated B cells and plasma cells in MRL/lpr mice. Splenic and lymph node mononuclear cells were isolated and stained with the indicated fluorescent antibodies. The frequencies of CD19^+^CD25^+^ or CD19^+^CD69^+^ activated B cells and plasma cells were quantified by flow cytometry. **(A, B)** Representative flow cytometry charts. **(C–E)** Quantitative analysis of the frequency of lymph node activated B cells and plasma cells. **(F–H)** Quantitative analysis of the frequency of splenic activated B cells and plasma cells in mice. The data are representative flow cytometry charts or expressed as the mean ± SD from each group (*n* = 5, 6) of mice and were analyzed by one-way ANOVA. *p < 0.05, **p < 0.01.

To understand the therapeutic action of QNZ, B cell activation and plasma cell differentiation were analyzed in the different groups of mice. Compared with the healthy controls, significantly higher percentages of lymph node and splenic CD19^+^CD25^+^ or CD19^+^CD69^+^ activated B cells as well as plasma cells were detected in the vehicle-treated MRL/lpr mice (Figure 5). In contrast, the percentages of lymph node and splenic CD19^+^CD25^+^ or CD19^+^CD69^+^ activated B cells as well as plasma cells in the QNZ- or FK-506-treated mice were significantly reduced, compared with the vehicle-treated MRL/lpr mice. Clearly, treatment with QNZ, like FK-506, inhibited the spontaneous activation of B cells and their differentiation into plasma cells in SLE-prone MRL/lpr mice.

### QNZ reduces the levels of serum IgG, ANA, and anti-dsDNA autoantibodies in MRL/lpr mice

**FIGURE 6:**
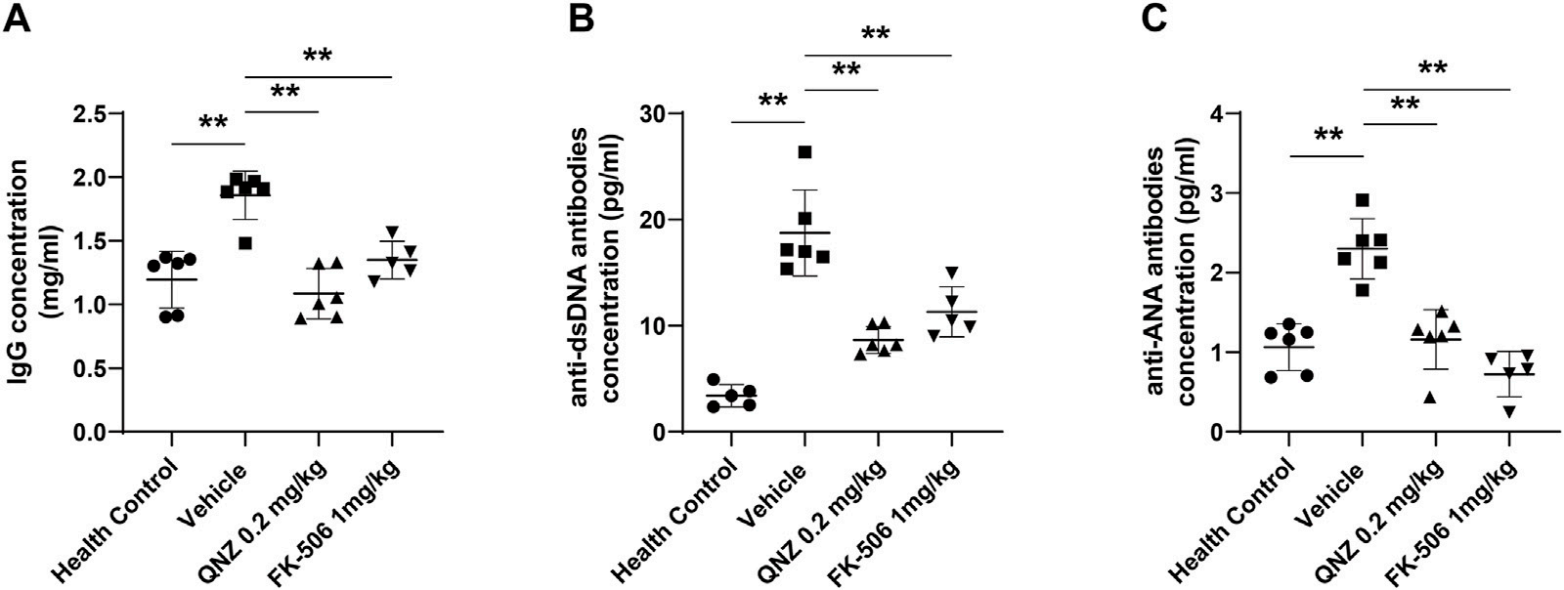
The levels of serum IgG, anti-dsDNA and ANA in mice. The levels of **(A)** serum IgG, **(B)** anti-dsDNA and **(C)** anti-ANA in individual mice were measured by ELISA using specific kits. Data are expressed as the mean ± SD of each group (*n* = 5–6) of mice. *p < 0.05, **p < 0.01.

The increase in the levels of serum autoantibodies is one of the most important characteristics of SLE, including IgG, anti-ANA, and anti-dsDNA. Finally, we measured the levels of serum anti-dsDNA, ANA autoantibodies and total IgG in individual mice by ELISA. Compared with the healthy controls, the levels of total IgG, ANA and anti-dsDNA significantly increased in the vehicle-treated MRL/lpr mice, which were significantly reduced in the QNZ- or FK-506-treated mice (Figure 6). Therefore, treatment with QNZ, like FK-506, significantly decreased the levels of serum total IgG and ANA and anti-dsDNA in MRL/lpr mice.

## Discussion

Current SLE therapies are still suboptimal because of their significant side effects. Over the last decades, B cell hyper-activation through the NF-κB signaling has been recognized to be critical for the pathogenesis of SLE. Previous studies have shown that some drugs, including curcumin, ibrutinib and losartan, can effectively inhibit rheumatoid arthritis, LN, systemic sclerosis and other autoimmune diseases by attenuating the NF-κB signaling to improve B cell function (Huang et al., 2013; Li et al., 2019; Wang et al., 2019; Einhaus et al., 2020). To understand the potential mechanisms underlying the action of QNZ, a NF-κB inhibitor, in B cell function, we tested the effects of different concentrations of QNZ on B cell function *in vitro*. We found that treatment with different doses of QNZ inhibited B cell activation, and antigen presentation-related molecule expression in a dose-dependent manner. Furthermore, treatment with QNZ also decreased the frequency of spontaneously activated B cells and plasma cells in MRL/lpr mice, accompanied by mitigating the SLE activity. To the best of our knowledge, this was the first report that QNZ treatment inhibited B cell activation and mitigated the severity of SLE. Conceivably, B cells and the NF-κB signaling may be valuable therapeutic targets for the intervention of SLE. Therefore, our findings may aid in the design of new therapies for SLE.

It is well known that aberrant autoreactive B cell responses are crucial for the development and progression of SLE. Functionally, activated B cells not only are important for humoral responses, but also for antigen presentation to induce T cell immunity through up-regulating the expression of co-stimulators, such as CD80, CD86, CD83, CD40, and MHC-II molecules. More importantly, enhanced B cell antigen presentation activity is crucial for the development and progression of autoimmune diseases, such as experimental autoimmune encephalomyelitis (EAE) and type 1 diabetes, because activated B cells can effectively present antigen determinants to T cells (Parker Harp et al., 1950). Indeed, CD80 or CD86 deficiency causes antigen-specific T cell anergy in mouse model of EAE (Chang et al., 2003; Kala et al., 2010), and MHC-II deficiency in B cells fails to induce EAE (Parker Harp et al., 1950). The defective CD80/86 expression in B cells prevents the development of proteoglycan-induced arthritis (O'Neill et al., 1950). Given that the balance of CD4^+^ T cell responses and regulatory T cells (Tregs) is crucial for maintaining peripheral T cell tolerance and control of autoimmune responses, the function of professional antigen presenting cells, including B cells, is important for inducing inflammatory autoimmune responses and maintaining the immunosuppressive activity of Tregs (Byun et al., 2014). Moreover, high levels of co-stimulators are expressed by activated B cells in pemphigus skin lesions (Zhou et al., 2020). Moreover, follicular helper T (Tfh) cells represent a highly specialized subset of CD4^+^ T cells which are of paramount importance for the establishment of germinal centers (GCs). These cells provide critical signals to B cells, promoting the production of high-affinity antibodies, class switching, and the development of long-term memory (Pedros et al., 2018). Mature Tfh cells are induced by interactions with homologous B cells mainly at the T-B border (Crotty, 2014). NF-κB-induced kinase (NIK) is a noncanonical NF-κB pathway required for Tfh cell development. NIK fulfills a pivotal role in the development of antigen-stimulated Tfh cells, and its function is not intrinsic to T cells, but rather mediated by the regulation of B cell support (Walsh et al., 2015). Consequently, the inhibition of NF-κB activation might result in the suppression of Tfh differentiation and expansion, thereby hampering GC development and the generation of plasma cells. Moreover, the inhibition of NF-κB activation might also lead to the suppression of B cell gene and function, thereby negatively affecting the interaction between Tfh and B cells. Thus, the B and T cell interaction may determine the development and progression of SLE. In this study, we found that QNZ treatment significantly mitigated the expression levels of co-stimulators on activated B cells. These decreased co-stimulator expressions on activated B cells should attenuate their antigen presentation activity, mitigating the severity of SLE in mice. Therefore, targeting the expression of these co-stimulators on antigen presenting cells and their antigen presenting activity may be valuable for control of SLE. We are interested in further investigating whether QNZ treatment can really minimize the antigen presenting activity of activated B cells during the process of SLE.

**FIGURE 7:**
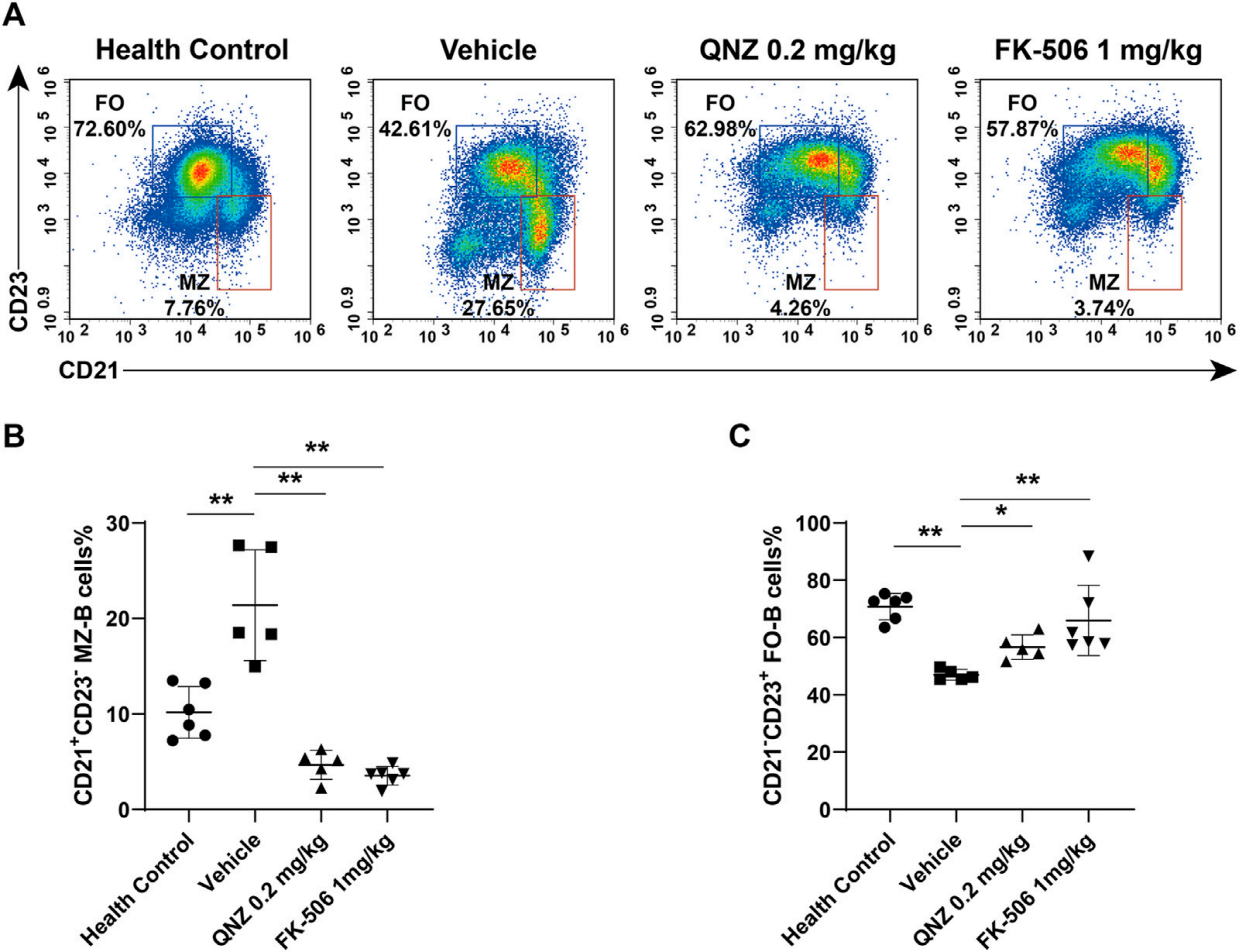
Flow cytometry analysis of the frequency of MZ-B cells and FO-B cells in MRL/lpr mice. **(A)** The frequency of splenic MZ-B cells (CD19^+^CD21^+^CD23^−^) and FO-B cells (CD19^+^CD21^−^CD23^+^) in mice. **(B, C)** Statistical analysis of the proportion of spleen MZ-B cells (CD19^+^CD21^+^CD23^−^) and FO-B cells (CD19^+^CD21^−^CD23^+^). The data are representative flow cytometry charts or expressed as the mean ± SD from each group (*n* = 5–6) of mice and were analyzed by one-way ANOVA. *p < 0.05, **p < 0.01.

Different subsets of B cells share similar roles in autoimmune diseases. Mature splenic B cells can develop into CD19^+^CD21^−^CD23^+^ follicular B cells (FO-B cells) and CD19^+^CD21^+^CD23^−^ marginal zone B cells (MZ-B cells) (Peeva et al., 2003). A previous study has shown that high frequency of activated B cells, plasma cells and MZ-B cells and high levels of autoantibodies are associated with serious kidney immune complex deposition in SLE mice (Grimaldi et al., 1950). Mature autoreactive B cells in estrogenic milieu are more likely to develop into MZ-B cells (Jeganathan et al., 2014). It is well known that B cell activating factor in the TNF family (BAFF) contributes to the pathogenesis of SLE. The TLR-activated innate MZ-B cells can produce autoantibodies in BAFF transgenic mice (Fletcher et al., 2011) while the loss of MZ-B cells delays the onset of LN (Fairfax et al., 2015). Therefore, MZ-B cells may be more important factors for the development of autoimmune diseases. During the process of SLE, the number of autoreactive MZ-B cells increase and activated MZ-B cells are important for T-independent immune responses by secreting anti-dsDNA *in vivo* (Schuster et al., 2002; Grimaldi, 2006). In contrast, therapeutic alleviation of SLE activity can reduce the frequency of MZ-B cells (Wang et al., 2020). Similarly, IL-6 can enhance B cell activity by increasing the production of anti-dsDNA in SLE patients while treatment with anti-IL-6 to ameliorate the SLE activity also reduces B cell proliferation induced by anti-CD40 and decreases the frequency of MZ-B cells (Liang et al., 2006). We found that treatment with QNZ, like FK-506, significantly decreased the frequency of splenic MZ-B cells in MRL/lpr mice (Figure 7). The decreased frequency of MZ-B cells by QNZ treatment may contribute to the decrease in the percentages of activated B cells and levels of serum anti-dsDNA in MRL/lpr mice.

The NF-κB is an important transcription factor in the induction of B cell immune responses. Numerous studies have suggested that constitutive NF-κB activation contributes to SLE pathogenesis, however, the loss of this transcription factor led to amelioration of many classical features of autoimmune disease. Evidently, aberrant cytokine signaling, such as the BAFF-TACI and CD40 signaling and high levels of NF-κBp65 phosphorylation are detected in autoreactive B cells of MRL/lpr mice and SLE patients (Dörner and Putterman, 2001; Stohl, 2003; Zhang et al., 2015). Engagement of these receptors on B cell can promote B cell activation and proliferation. In addition, engagement of TLR on B cells also can activate the NF-κB signaling and pro-inflammatory cytokine production (Ji et al., 2018). This proinflammatory cascade is crucial for the development and progression of SLE. Our data indicated that treatment with QNZ to inhibit the NF-κB signaling and attenuate the severity of SLE suggests that the NF-κB signaling may be a therapeutic target for intervention of SLE.

These data may provide new insights into the importance of B cells in the development and progression of SLE. It would be interesting to identify the molecular mechanisms underlying the action of QNZ in inhibiting B cell activation and SLE progression, which may help further elucidate the pathogenesis of SLE and aid in design of new, safer and more effective drugs, ultimately benefiting SLE patients.

## Conclusion

Our data indicated that treatment with QNZ mitigated B cell hyperactivation and plasma cell differentiation as well as the expression of antigen presenting-related molecules. More importantly, treatment with QNZ significantly attenuated the severity of SLE and LN in MRL/lpr mice by reducing the frequency of activated B and plasma cells and the levels of autoantibodies. Therefore, our findings may provide new insights into the importance of B cells in the development and progression of SLE and aid in the design of new therapies for SLE. We are interested in further clarifying how QNZ treatment modulates different subsets of B cells and inhibits SLE activity in the future studies.

## Data Availability

The original contributions presented in the study are included in the article/Supplementary Materials, further inquiries can be directed to the corresponding authors.

## Abbreviations

SLE, systemic lupus erythematosus; H&E, hematoxylin and eosin; LN, lupus nephritis; Bregs, regulatory B cells; NF-κB, nuclear factor kappa B; TLR, Toll-like receptor; PF, paeoniflorin; CFSE, 5(6)-carboxyfluorescein diacetate succinimidyl ester; DMSO, dissolved in dimethyl sulfoxide; FBS, fetal bowel serum; SPF, specific pathogen-free; CCK-8, cell counting kit-8; MZ-B cells, marginal zone B cells; FO-B cells, follicular B cells; OCT, optimum cutting temperature; Tregs, regulatory T cells.
